# Thermoluminescence properties and new insights on the UV-vis absorption features of colorless quartz after γ-ray irradiation

**DOI:** 10.1039/d4ra03116d

**Published:** 2024-06-17

**Authors:** Tran Ngoc, Nguyen Xuan Ca, Nguyen Trong Thanh, Nguyen Manh Hung, Pham Tien Du, Tran Thi Chung Thuy, Nguyen Thi Huong, Phan Van Do

**Affiliations:** a Institute of Research and Development, Duy Tan University Da Nang 550000 Vietnam; b Faculty of Natural Sciences, Duy Tan University Da Nang 550000 Vietnam; c Institute of Science and Technology, TNU-University of Sciences Thai Nguyen Viet Nam; d Institute of Materials Science, Vietnam Academy of Science and Technology Hanoi Vietnam; e Thuyloi University 175 Tay Son, Dong Da Hanoi Vietnam phanvando@tlu.edu.vn

## Abstract

In this paper, we present the results of research on the thermoluminescence (TL) and optical absorption (OA) properties of colorless natural quartz (including natural quartz samples, sodium ion (Na^+^) rich samples (by diffusion), and alkali metal (M^+^) ion poor samples (by sweep)). In detail, the relationship between the TL glow peaks and the emission wavelength was determined. The dynamics parameters (*E*_T_, *s*, *τ*) have been computed for all TL peaks on the glow curve. The recombination mechanism electron–hole with the participation of the region energy has been determined for all electron traps in the temperature range of 50–430 °C through thermally stimulated conductivity measurement (TSC). Nonlinearity and approaching signal saturation are observed at doses above 22 Gy for the electron trap at 110 °C, above 45 Gy for the electron trap at 238 °C, and 80 Gy for the electron traps at 325 °C and 375 °C. The role of irradiation and heat treatment in the formation of absorption centers as well as the relationship of these centers to electronic traps have been also investigated in detail. The role of M^+^ ions and hydrogen ions (H^+^) for the absorption bands in the UV-vis region has been discussed. The results of the combination of the TL measurement and monochromatic light absorption according to temperature show that the TL process occurs concurrently with the reduction of the absorbent center produced in the irradiation process.

## Abstract 2

3-D TL spectrum (a) and contour plot (2D-TL) of the emission spectrum (b) of the natural quartz (with the heating rate of *β* = 5 °C s^−1^, γ-ray irradiation Dγ = 6.4 Gy).

## Graphical abstract

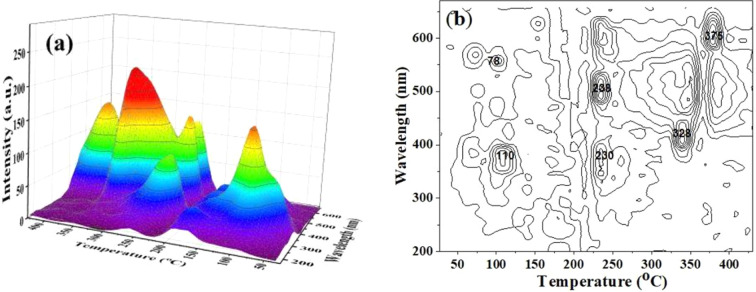

## 1. Introduction

Quartz is one of the minerals that accounts for a large proportion of the Earth's crust.^1^ Unlike other minerals, quartz usually exists in a single-crystal form. Compared to other minerals, quartz has a fairly stable crystal structure and is a fairly clean mineral with few foreign impurities, but that does not mean absolute purity. Natural quartz crystals contain quite a lot of impurities, even though the concentrations are quite low, such as Al, Fe, Ge, Na, Li, K, *etc.* These impurities create defects in their crystal lattice structure.^2–5^ The presence of defects gives rise to localized energy states that are distributed in the forbidden region of the crystal. These energy states play the role of electron or hole traps. Therefore, quartz can strongly absorb some kinds of ionizing radiation and record radiation information as a latent signal within its crystal lattice. This information may be erased by certain events, such as daylight exposure and/or heat. Consequently, quartz can be used as a natural dosimeter for quantifying the irradiation history of a material.^6–9^ There are two ways to obtain a latent signal from the crystals of quartz, which are thermoluminescence (TL) and optically stimulated luminescence (OSL) or the combination of both ways. Both of these signals result from the measurement of trapped electrons held within the crystals *via* the light emitted by quartz when it is exposed to light or heat.^1,10^ Because the thermodynamic conditions of the formation environment greatly influence the composition of defects and internal structure of quartz crystals, the structure of traps and centers in quartz being formed in several different geological settings is different, which is shown in the shape and maximum position on their glow curves. On the other hand, when quartz is affected by radiation, new defects can also be formed and the properties of the trap centers may be changed. Research results all show that pure natural quartz (SiO_2_) has a rather large optical band gap, so it cannot absorb photons with wavelengths greater than 200 nm.^4,11^ Absorption bands of light in the visible (Vis) and near-ultraviolet (UV) regions appear only after the quartz is irradiated by ionizing radiations (such as X-rays, γ-rays, or neutron irradiation). The intensities of these bands increase with the dose and they can be removed by heat treatment in the air to 500 °C. The absorption of ionizing radiation energy can affect the luminescence as well as the color of colorless natural quartz. Therefore, natural colorless quartz can change color when subjected to irradiation and heat treatment.^11–13^

In a previous paper, we described the role of sodium ions in the TL peaks and the effect of blue light (450 nm) on electron trapping in natural quartz irradiated in the laboratory.^9^ In this paper, we present the results of research on thermoluminescence (TL) and optical absorption (OA) properties by a combination of TL, OA measurement techniques, and TSC on colorless natural quartz of Vietnam. In 2012, M. Singh *et al.* reported the study results on the TL characteristics of high gamma dose irradiated natural quartz.^14^ In this paper, the authors investigated the dose response of the TL intensity, the temperature change of two TL peaks (bands in the region of 49–499 K and 634–666 K), and the generation/destruction of the defects. The results were obtained by comparing the TL luminescence curves of samples irradiated with different doses. The irradiation was carried out by gamma-ray with very high doses (30–280 kGy). In addition, the kinetic parameters of the independent peaks were also determined using R. Chen's empirical formula.^15^ For our work, we also studied the dose response in the region of low doses (0–10 kGy). The kinetic parameters of all TL peaks in the range of 27–420 °C (peaks at 78, 110, 230, 325 and 375 °C) were also calculated using the partial thermal-cleaning technique. However, these are not the main results that we want to achieve. Our attention is focused on four purposes: (i) the mechanism for capturing and releasing the charge carriers during the TL process; (ii) the role of irradiation and heat treatment in the formation of absorption centers as well as the relationship of these centers to electronic traps; (iii) the role of the Na^+^ and H^+^ ions in the UV-vis absorption properties of laboratory-irradiated colorless natural quartz; and (iv) the correlation between the OA and TL processes with bleaching time by blue light.

## 2. Experimental methods

The samples of colorless sedimentary quartz crystals of different sizes from different caves of ancient limestone blocks of the carbon-Permian age in the world natural heritage site Phong Nha – Ke Bang National Park (VietNam) (17°21′N, 105°46′E) were used in this study. Natural quartz crystals are collected from invasive pegmatite rock in altered rock zones running vertically and diagonally along the deep cliffs into the cave. Some other samples were collected from sandy limestone deposit rock blocks located in an underground stream inside the cave. Because the sampling location is deep inside the cave, daylight cannot reach at all, and the average annual temperature is about 13–15 °C. Samples are then placed in a black plastic bag until they are processed in the laboratory. The steps of sample processing were carried out under red light. The blue light was only intentionally irradiated onto the sample for the bleaching measurements. The corrosive solvents such as HF (0.1 M), and HCl (0.1 M) were used to clean the crystal surface and dissolve other symbiotic minerals. The sample was then divided into two groups depending on the research purpose. Small-sized crystals are crushed and mixed to ensure uniformity (the particle size is around 100–130 μm). This powder was used for the measurements of TL and XRD. Larger crystals are cut into sheets of 5 × 5 × 1 mm^3^ and polished on both sides with aluminum oxide powder used in absorbance measurements. The sample preparation process was treated under subdued red light to avoid the fading of charges in the TL trap due to irradiation in the natural environment. In the TSC measurement, we used two electrodes of silver paint that covered the entire two faces of the sample of 10 × 5 × 3 mm^3^. Absorption spectra were recorded with a Cary 50 instrument (UV-vis, Varian, USA). The 345 and 450 nm light used in the bleaching and absorption measurements were taken from a xenon lamp (XBO-100 W) through a monochromator SPM2 using a grating of 1302 lines or using the region filters *Φ*C6 (320–450 nm), CC5 (340–500 nm) and 3C7 (520–750 nm). The measurements of integral TL curves were performed with the Hashaws TLD-5500 system (USA). 3-D TL spectrum was measured by a TL spectrometer using a fast scanning step motor with the scanning speed of 12 nm s^−1^ with a very narrow temperature range and heating rate of 5 °C s^−1^. TL spectra measurements were performed using a Fluorolog-3 Model FL3-22, resolution of 0.3 nm, excitation by light from xenon lamps 100 W. The γ-ray irradiation process was carried out at room temperature using a Cobalt PICKER/C9 source with a dose rate of 32 cGy minute^−1^.

## 3. Results and discussion

### 3.1. Sample analysis

**Fig. 1:**
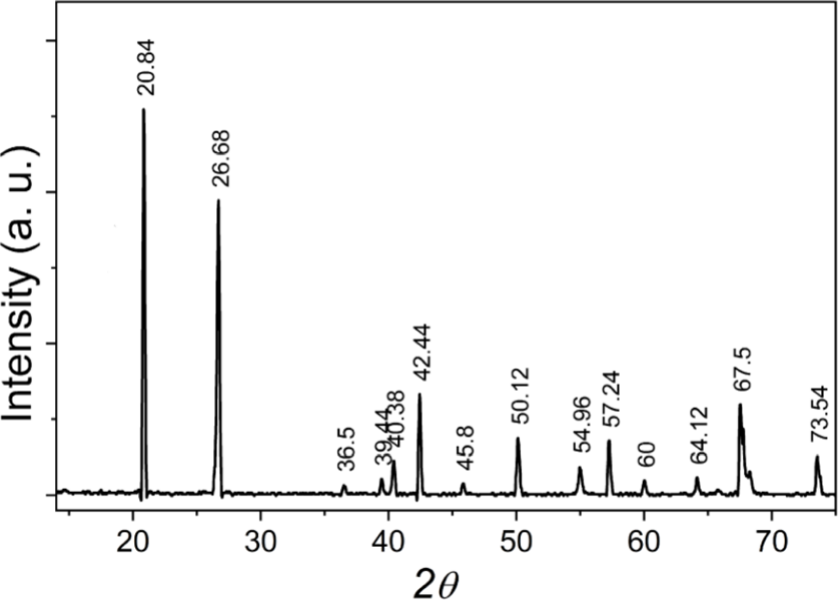
X-ray diffraction pattern of natural quartz crystal in the 2*θ* range from 10 to 80°.

X-ray diffraction measurements were performed on a Siemens-D5000 (Germany). Analysis of the X-ray diffraction pattern of the natural quartz sample in Fig. 1 shows that the sample is highly clean, with no phases of other impurities appearing. The crystal has a hexagonal structure, quantities such as lattice constant and density have been determined with *a*_o_ = 4.903 Å, *c*_o_ = 5.408 Å and *δ* = 2.652 g cm^−3^. The composition of impurities contained in natural quartz samples was determined by microzone analysis – scanning electron microscope on CAMEBAX analysis system (France) with integrated EDX (Energy Dispersive X-ray) detector (beam size 0.01 μm, area as small as 0.5 μm^2^, the sensitivity can reach 0.01%), results in Table 1. This result shows that the concentration of impurities is not large but quite diverse, and they are origin creates defects in the crystal lattice structure of natural quartz.

**Table 1:** Composition of impurities in colorless natural quartz

Element	SiO_2_ (%)	Al_2_O_3_ (%)	CaO (%)	FeO (%)	TiO_2_ (%)	Other elements (%)
Content	99.84	0.08	0.02	0.03	0.02	0.01

### 3.2. The thermoluminescence properties

#### 3.2.1. The TL glow curves and TL spectra

**Fig. 2:**
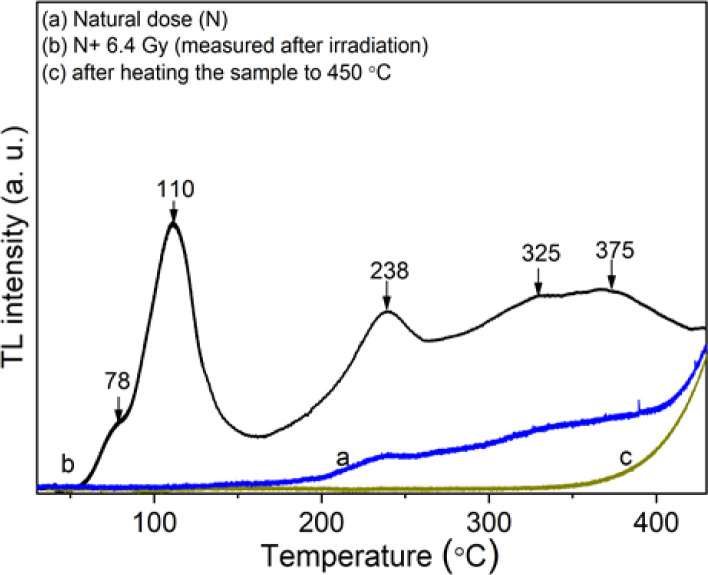
The TL glow curve of the natural quartz in the region of the temperature from RT to 430 °C with the heating rate of *β* = 5 °C s^−1^, γ-ray irradiation (Dγ = 6.4 Gy).

The TL glow curves, which were recorded in the region of the temperature from room temperature (RT) to 430 °C with a heating rate of *β* = 5 °C s^−1^, were presented in Fig. 2: curve (a) of the natural sample (N) (without additional irradiation), curve (b) of the natural sample irradiated with dose supplement (N + 6.4 Gy) of γ-ray (measured immediately after irradiation), and curve (c) of the natural sample after being measured the TL to get curve b. To obtain curve c, the natural quartz sample was irradiated by γ-ray (6.4 Gy), and then the TL measurement was carried out on this sample from RT to 430 °C to get curve b. Next, the sample was cooled to room temperature, then the TL measurement was performed a second time from RT to 430 °C to obtain the curve c.

It can be seen that on curve (a), only glow peaks in the temperature range above 200 °C appear, but the signal intensity is quite small because the sample was subjected to only natural dose irradiation (N) without additional laboratory irradiation. Curve (b) consists of glow peaks appearing in the temperature ranges of 50–150 °C (*T*_max_ at 110 °C), 160–280 °C (*T*_max_ at 238 °C), and 280–400 °C (*T*_max_ at 325 °C and 375 °C). The glow peaks no longer appear when the sample is heated to 450 °C (curve (c)), this means that all the charge traps in this temperature range are empty. We see that the glow curve structure of natural quartz is quite complex with the appearance of many overlapping glow peaks in the temperature range from 50–400 °C. However, like the published results on the TL properties of natural quartz in many places around the world, the main glow peaks correspond to the *T*_max_ around 110 °C, 240 °C, 325 °C and 375 °C all appear on the glow curve of the samples.^2,15^

**Fig. 3:**
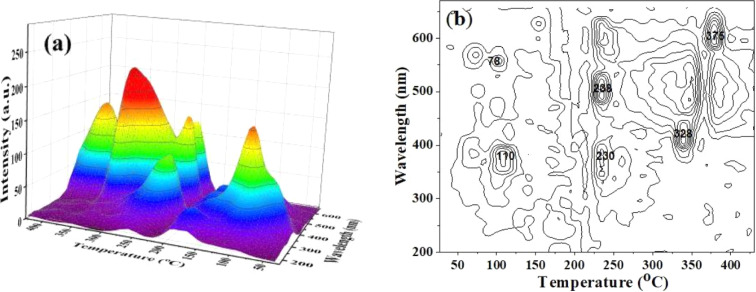
3-D TL spectrum (a) and contour plot (2D-TL) of the emission spectrum (b) of the natural quartz (with the heating rate of *β* = 5 °C s^−1^, γ-ray irradiation Dγ = 6.4 Gy).

Fig. 3 shows the 3-D (a) and 2-D (b) TL spectrum recorded in the temperature range from RT to 430 °C and the wavelength range from 200 nm to 700 nm with the heating rate of *β* = 5 °C s^−1^, the sample was previously irradiated with 6.4 Gy dose of gamma. Analyzing the obtained spectrum along the wavelength axis, we see that three main emission bands are of most interest including 300–430 nm (*λ*_max_ = 380 nm), 440–550 nm (*λ*_max_ = 470 nm), and 550–630 nm (*λ*_max_ = 590 nm). The relationship of these radiation bands with the electron traps is shown in Fig. 3b. Based on Fig. 3b, we found that the TL spectra include three bands: 300–430 nm (*λ*_max_ = 380 nm), 440–530 nm (*λ*_max_ = 470 nm), and 550–630 nm (*λ*_max_ = 470 nm). (i) The first band is related to most of the electron traps corresponding to temperatures around 110 °C, 238 °C, and 325 °C. The origins of this emission band can be expressed as follows: the electrons were generated during irradiation can be captured at the electron traps (which are lattice defects in the SiO_4_^−^ structure). During the heating process, they were released from the traps and then recombined with a hole at the [H_3_O_4_]^0^ center. This recombination process is accompanied by the emission of photons.^9,16–21^ (ii) The second one is related to electron traps that give rise to a signal above 200 °C in the TL glow curve. The signal spectrum is relatively wide and asymmetric between two sides of the short and long wavelengths. Thus, this spectrum is also asymmetric on the energy axis. The generation process of this band in the TL spectrum was interpreted the same as that of the 300–430 nm emission band, in which the position due to impurity ions replaces Si and binds to alkali metal ions act as electron traps (*e.g.* [TiO_4_/M^+^], [[GeO_4_]/M^+^]) and recombination center (*e.g.* [AlO_4_]^0^) act as hole traps.^9,16,17,19,21–23^ (iii) The last band is related to traps with temperatures from 200 to 280 °C, and above 350 °C. The spectral intensity in this band is usually much less intense than other emission bands. The origin of the 550–630 nm band is explained similarly to the above two cases. However, this band is related to the [TiO_4_/M^+^], [GeO_4_]/M^+^], [SiO_4_/h^+^] electron traps and the [SiO_4_/M]^+^, E′ hole centers.^2,16,22,24–28^

As shown in Fig. 3b, when the temperature increases, the *T*_max_ does not show a specific trend of red or blue spectral shift. Instead, a complex combination of red and blue shifts is observed. This behavior in the TL spectrum can be explained based on the TL theory.^1,9^ While the sample is heated, electrons released from an electron trap can recombine with holes at different recombination centers to fluoresce. The wavelength of the emitted light depends on the nature and position of the recombination center but is independent of the temperature of the sample. So, in this case, a spectral shift (red or blue shift) will not be observed according to any specific rule.

#### 3.2.2. Determine the kinematic parameters

**Fig. 4:**
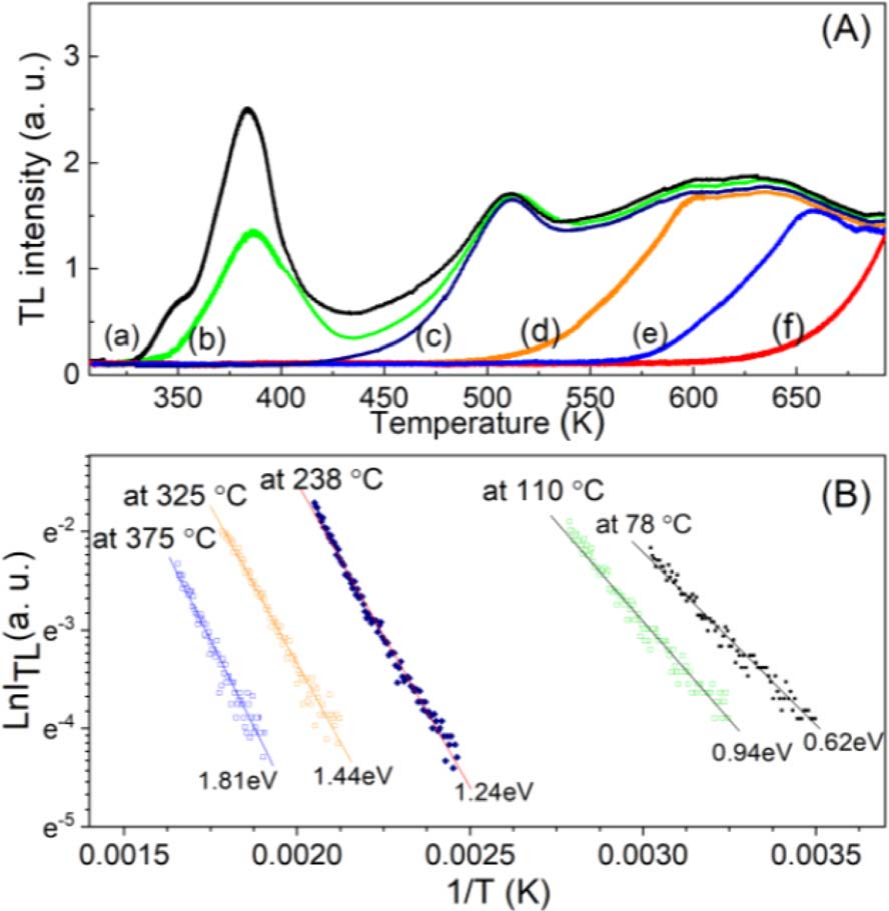
(A) The obtained TL glow curves using the partial thermal-cleaning technique with *T*_stop_ (a) 30 °C, (b) 60 °C, (c) 110 °C, (d) 230 °C, (e) 320 °C and (f) 380 °C. (B) The graph of ln *I* with 1/*T* corresponds to 15% of the initial rise part of the TL curves of the low-temperature peaks (in (A)).

Since the TL glow curve has a complex structure with many overlapping emission bands, to calculate the kinetic parameters for the respective charge traps, we applied the initial rise method. Using the partial thermal-cleaning technique, heating the sample to the maximum of the first peak (*T*_stop_) causes the trap corresponding to this temperature to empty. This experiment is performed according to the following steps: a batch of powder samples (∼1.0 g) with a particle size of 100–130 μm was heat treated at 500 °C, and then was irradiated by γ-ray with a dose rate of 6.4 Gy. Each measurement used 10 mg of the irradiated sample. This sample was put into the measurement tray, and the Hashaws was set in the preheat mode at a pre-selected temperature (*T*_stop_). After being preheated, the TL measurement was carried out from RT to 430 °C. Thus, the samples used in this experiment were heat-treated and irradiated to 6.4 Gy before being measured. For this method, the low-temperature peak can be eliminated, and the initial rise part of a successive peak can be obtained by subsequent TL measurement.^15,29^ Because the initial rising part of a thermoluminescence peak depends on temperature by the expression *I*_TL_(*T*) = *B* exp(−*E*_T_/*kT*), in which *I*_TL_(*T*) is the TL intensity at temperature *T*, the *B* constant is almost independent of temperature, *E*_T_ is the electron trap depth, *k* is the Boltzmann constant and *T* is the temperature, the thermal activation energy can be determined from this one. Taking the initial increase part on each curve corresponding to 15% of the maximum intensity (in Fig. 4A) and plotting the dependence of ln(*I*_TL_) *versus* 1/*T* (the “Arrhenius plot”) at the various values of *T*_stop_ (Fig. 4B), then the thermal activation energy is determined through the slope of this graph.

**Fig. 5:**
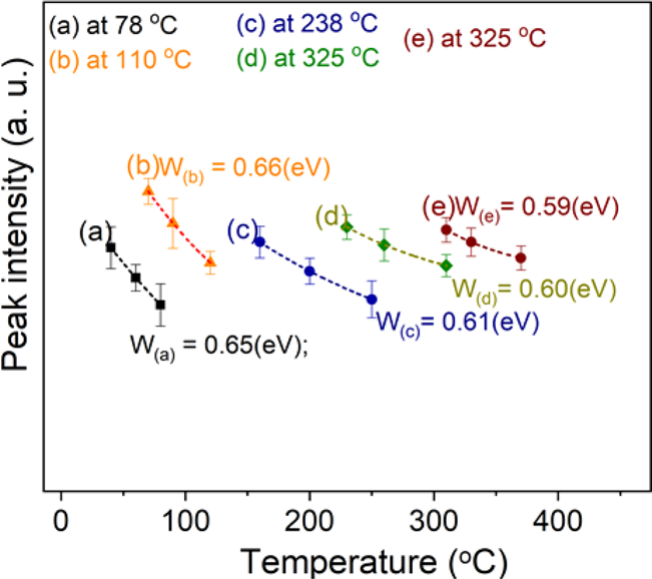
The decrease in TL signal intensity (at the maximum temperature *T*_max_) of the glow peaks corresponding to the emission center at 380 nm, 470 nm or 590 nm.

It is well known that the initial rise technique is affected by thermal quenching so the *E*_T_ value determined by this method is usually lower than the correct value: *E*_T_ = *E*^(q)^_T_ − Δ*E*_T_. It is possible to correct the values of the *E*_T_ derived with the initial rise technique using the thermal quenching parameters *η*(*T*) = 1/(1 + *C* e^−*W*/*kT*^), then the additive term Δ*E*_T_ is the correction by the formula: Δ*E*_T_ = *W*/[1 + *C* e^−*W*/*kT*^]^−1^ (the value of Δ*E*_T_ is a function of temperature and is always less than *W*). Experimentally, by monitoring the decrease of the signals TL with the increase of the heating rate (*i.e.* with the shift of the maximum temperature) of peaks with the same recombination center, a value of *W* can be evaluated. Fig. 5 shows the result of monitoring the decrease in TL signal intensity (at the maximum temperature *T*_max_) of the glow peaks corresponding to the emission center at 380 nm, 470 nm, and 590 nm. For this experiment, after the samples were irradiated with a gamma dose of 6.4 Gy, the TL measurements were performed with different heating rates of 3.0 °C s^−1^, 5 °C s^−1^, and 7 °C s^−1^. The value of *W* can be evaluated by nonlinear curve fit by the function ExpDec1. For our natural quartz samples, the *W* values are found to be 0.65 eV for peaks with *T*_max_ temperatures below 200 °C (with the same recombination center, emission at 380 nm) and 0.60 eV for peaks with *T*_max_ temperature above 200 °C (with the same recombination center, emission at 470 nm and 590 nm). Substituting the *W* values into the above formula, the Δ*E*_T_ deviations were found to be 0.27 and 0.21 eV for peaks with *T*_max_ temperatures below 200 °C and above 200 °C, respectively.

**Fig. 6:**
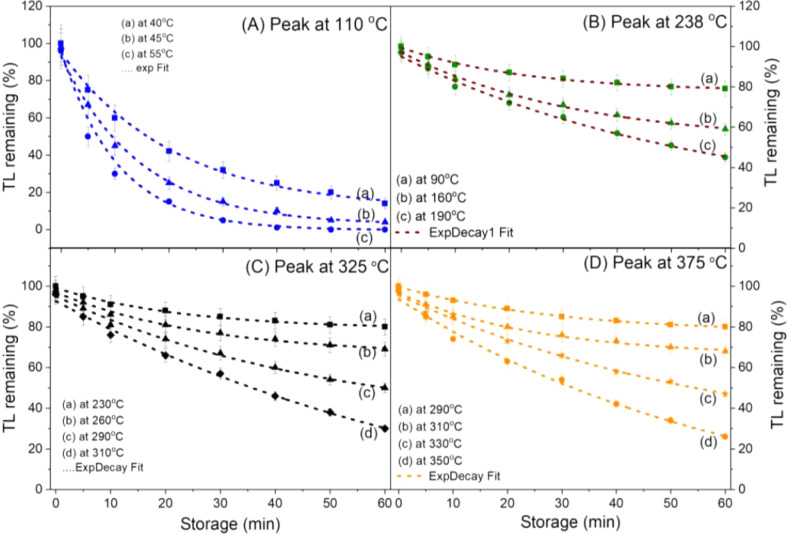
The isothermal decay of glow peaks at different storage temperatures: (A) peak at 110 °C, (B) peak at 238 °C, (C) peak at 325 °C and (D) peak at 375 °C.

The lifetime (*τ*) of the glow peaks can be determined by the isothermal decay method. If the condition of first-order kinetics is verified, the decay of the TL signal at a constant temperature is given by: *I*_1_ = *I*_0_ exp(−*t*_1_/*τ*_1_), in which *I*_0_ is the TL intensity of the glow peak at temperature *T*_0_, and *I*_1_ is the TL intensity when the sample is kept at temperature *T*_1_ for time *t*_1_. The lifetimes of the electron on the trap at temperatures of *T*_0_ and *T* are *τ*_0_ and *τ*, respectively. These quantities are calculated by the expression: *τ*_0_ = *s*_0_^−1^ exp(−*E*/*kT*_0_) and *τ*_1_ = *s*_1_^−1^ exp(−*E*/*kT*_1_), respectively. Since the frequency does not depend on the temperature, so *s*_0_ = *s*_1_, we have *τ*_0_ = *τ*_1_ exp[(*E*/*k*)(1/*T*_0_ − 1/*T*_1_)]. Therefore, by monitoring the decay of the TL signal at a constant temperature *T*, the corresponding lifetime *τ*(*T*) can be evaluated.^29–31^ Repeating with the temperatures *T*_2_, *T*_3_, *etc.*, we will have a curve showing the dependence of the lifetime (*τ*) on the temperature *T*. For low temperatures *T*_0_ (usually lower than RT) the *τ*_0_ value cannot be calculated directly, so this parameter was estimated by the interpolation method from the fitting curve according to the function ExpDec. The results monitoring the decay of the TL signal of main peaks at different temperatures are presented in Fig. 6. The obtained lifetimes for the peaks at different storage temperatures are presented in Table 2.

**Table 2:** The lifetime (*τ*) of the peaks at different storage temperatures (*T*)

*T* (°C)	*τ* of peak 78 °C	*τ* of peak 110 °C	*τ* of peak 230 °C	*τ* of peak 325 °C	*τ* of peak 375 °C
15	3.6^a^ (h)	34.6^a^ (h)	4.7 × 10^3^^a^ (year)	2.1 × 10^8^^a^ (year)	2 × 10^10^^a^ (year)
25	0.7^a^ (min)	7.6^a^ (h)	0.42 × 10^3^^a^ (year)	2.5 × 10^7^^a^ (year)	1.5 × 10^9^^a^ (year)
40	4.7 (s)				
55	1.7 (s)				
70	—	34.1 (min)			
80	—	3.7 (min)			
90	—	54.3 (s)	385.7 (h)		
160	—	—	320.4 (h)		
190	—	—	11.3 (h)		
230	—	—	6.4 (min)	4529.7 (h)	
260	—	—	—	106.5 (h)	
290	—	—	—	23.6 (h)	1157.9 (h)
310	—	—	—	3.8 (h)	128.7 (h)
330	—	—	—	—	31.6 (h)
350	—	—	—	—	21.5 (min)

a Extrapolated values.

The frequency factor (*s*) was also calculated by using the formula *s* = (1/*τ*)exp(*E*_T_/*kT*).^15^ It is also worth noting that the values of activation energies (*E*_T_) and lifetime (*τ*) determined from this method are often errors inevitable as described above. Since the values of the activation energy and lifetime are variables in the expression used to calculate (*s*), the obtained value of the frequency factor (*s*) is also biased. Physically, the frequency factor indicates the releasing possibility of the electrons trapped on a certain trap. Thus, if the error has the same order, the result is still acceptable. The values of kinematic parameters corresponding to each glow peak (after correction) are listed in Table 3.

**Table 3:** Aggregate the dynamics parameters (*E*_T_, *s*, *τ*) for the TL peaks of natural quartz (at 25 °C)

Peak (°C)	*E*_T_ (eV)	*τ*_(at RT)_ (min, h, year)	*s* (Hz)
78	0.89	0.7 min	3.3 × 10^14^
110	1.21	7.6 h	8.7 × 10^13^
238	1.45	0.42 × 10^3^ years	2.2 × 10^10^
325	1.65	2.5 × 10^7^ years	1.6 × 10^10^
375	2.02	1.5 × 10^9^ years	8.2 × 10^9^

It can be seen that the glow peak centered at 110 °C has a relatively short lifetime at room temperature (several dozen hours), and a very large frequency factor (of the order of 10^12^–10^13^ Hz), which is a group of shallow traps. For this reason, peaks in the 50–150 °C range do not appear at all on the glow curves of the additional non-irradiated samples (see curves (a) in Fig. 2). The glow peaks with the *T*_max_ at 238 °C, 325 °C, and 375 °C, which have an average lifetime (10^3^–10^6^ years) and a lower frequency factor (10^9^–10^10^ Hz), are assigned to deep traps. However, only the traps with the *T*_max_ at 230 °C and 375 °C are stable with temperature, light, and time conditions, while the trap with the *T*_max_ at 325 °C is said to be very bleached when the sample is exposed to daylight.

**Fig. 7:**
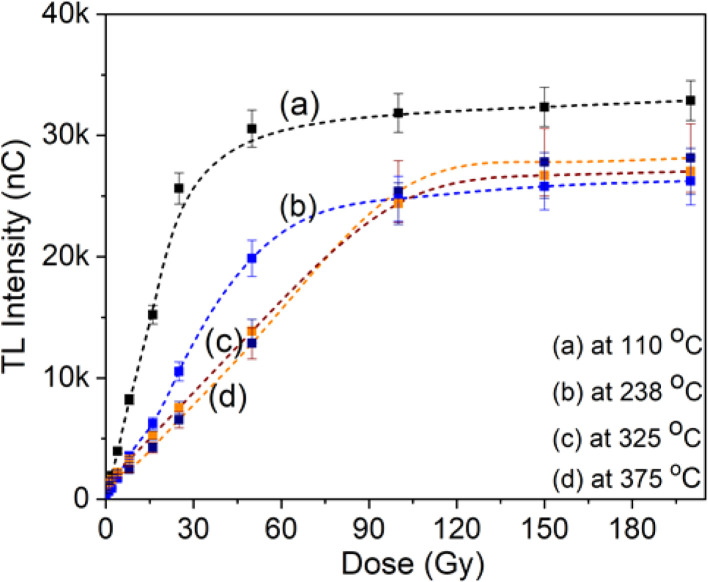
The TL signal intensity response of main peaks (110, 238, 325 and 375 °C) to gamma irradiation dose.

The TL signal intensity response at main peaks at 110 °C, 238 °C, 325 °C and 375 °C to gamma irradiation dose is shown in Fig. 7. The results show that the electron trap at a temperature of 110 °C has a linear response in the dose range from 0.05 Gy to 22 Gy. Signal saturation began to be observed at doses above 22 Gy. For the electron trap at 238 °C, saturation appears when the irradiation dose is above 45 Gy. These behaviors at bands 325 °C and 375 °C are observed at doses exceeding 80 Gy. For quartz, we are only interested in the linear region and especially the saturation limit on the response curve, since that is relevant to the application in TL dating. With the obtained results, we can conclude that quartz can be used for TL dating of sediments or building materials (including unfired building materials).

#### 3.2.3. Thermally stimulated conductivity

**Fig. 8:**
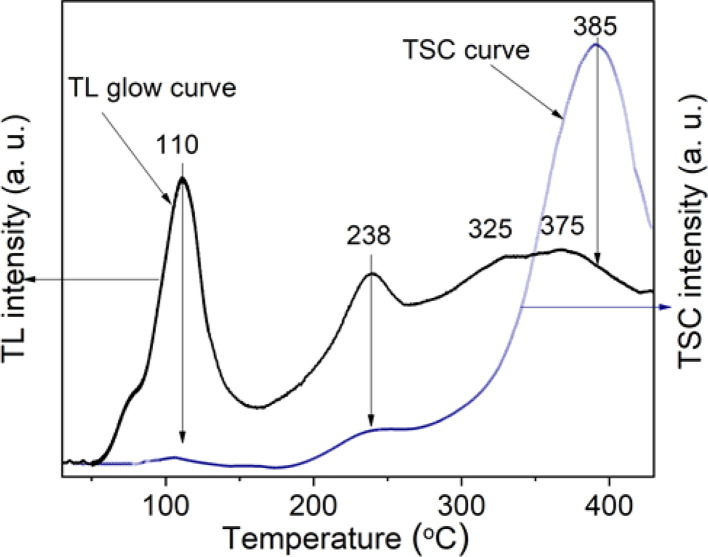
The TL glow curve and TSC curve (the samples are both γ-rays irradiated Dγ = 19.2 Gy, the heating rate of *β* = 5 °C s^−1^).

The kinetics of charge carriers during material irradiation and heating is an important issue in model selection and kinetic equations for the TL process. The obtained results from the combination of the TL and TSC measurements would be the clearest experimental evidence for the participation (or not participation) of the energy band in the kinetics of the carriers in electron traps after being released by heating. Fig. 8 presents the results of the TL glow curve and TSC measurements for the natural quartz samples with the same heating rate of 5 °C s^−1^. The two samples used for the TL and TSC measurements are natural quartz samples separated from a crystal (subjected to natural irradiation dose N). Before performing TL and TSC measurements, these samples were additionally irradiated with a laboratory dose of 19.2 Gy to fill the traps on the low-temperature side (below 200 °C). This would create a high sensitivity of the TL and TSC signals. For all traps in the temperature range of 50–400 °C, the result shows that the recombination process occurring in natural quartz has the participation of the energy bands (electrons move through the conduction and valence bands). This mechanism is dominant for the traps above 300 °C. As shown in Fig. 8, the peak position of the TSC curve is shifted towards the high temperature compared with the corresponding peak in the TL curve. These results are completely consistent with the theory of TL and TSC processes. Because of the superposition of the peaks on the TSC curve, it is difficult to evaluate the recombination probability at a recombination center involving a certain electron trap. Chen and McKeever^30^ reported that the recombination of electrons and holes includes two mechanisms: direct recombination, and recombination through the conduction band. In the second mechanism, after being released from electron traps, the flow of the electrons will move through the conduction band before recombining with a hole at the recombination center. The TL process includes both mechanisms whereas the TSC only involves the second one. Thus, the TSC process takes longer than the TL one. However, the issue we are interested in is the TSC curve shape and the location of the peaks, because that is the most convincing and specific experimental evidence to demonstrate the recombination mechanism with the participation of the energy band in the TL process.

**Fig. 9:**
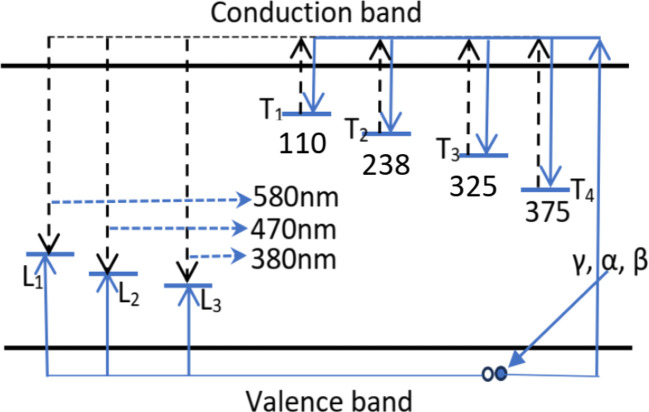
The energy band diagram for the kinetics of charge carriers in the TL process: T_1,2,3,4_ symbols are electron traps; L_1,2,3_ symbols are hole traps; solid arrows (→) indicate the movement of carriers during irradiation and dashed arrows (⇢) illustrate the carrier movement during heating.

The TL process occurring in quartz that we observe here can be illustrated by the energy band diagram in Fig. 9. According to the thermoluminescence theory, in materials with a wide bandgap (*e.g.* semiconductor or dielectric materials), defects such as lattice defects or impurity ions create energy levels distributed in the forbidden band of the crystal, and they are called the centers or the traps. The centers and traps located lower than the bottom of the conduction band but higher than the Fermium level tend to capture electrons when the material is ionized, so they are called the electron traps (T). The centers and traps lie higher than the top of the valence band but lower than the Fermium level have a tendency to capture the holes, they are called the hole centers. After being released from the traps, electrons can move to recombine with holes in the centers. This recombination is accompanied by light emission, so these centers are called luminescence centers (L centers). For colorless natural quartz, the study results of the TL process (including the TL glow curve and TL spectrum) show the existence of four T trap clusters (T_1_, T_2_, T_3_, and T_4_) corresponding to the temperature peaks of 110 °C, 280 °C, 325 °C and 375 °C, and three luminescence centers L_1_, L_2_ and L_3_ emit radiation at 380 nm, 470 nm and 500 nm. Thus, Fig. 9 indicates the energy band diagram for the kinetics of charge carriers in the TL process. The properties and the relationship between the emission bands and the TL glow peaks are presented in Table 4.

**Table 4:** The relationship between the emission bands and the TL glow peaks

*T*_max_ (°C)	Wavelength (nm)	Recombination mechanism
50–150 (*T*_max_ = 110) (photosensitive)	300–430 (*λ*_max_ = 380)	The participation of the energy band
160–280 (*T*_max_ = 238)	300–630 (*λ*_max_ = 380, 470, 580)	The participation of the energy band
280–350(*T*_max_ = 325) (photosensitive)	300–530 (*λ*_max_ = 380, 470)	The participation of the energy band (prevails)
350–400 (*T*_max_ = 375)	440–630 (*λ*_max_ = 470, 580)	The participation of the energy band (prevails)

### 3.3. Absorption properties in the UV-vis region of natural quartz

#### 3.3.1. The absorption spectra and the optical band gap

**Fig. 10:**
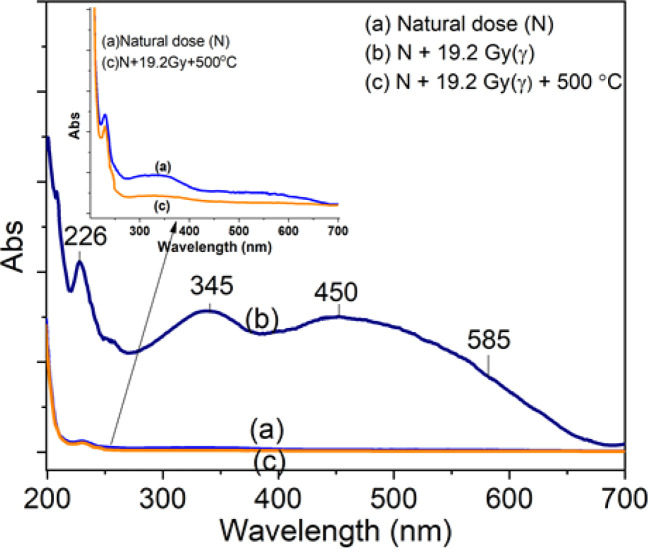
The absorption spectra of quartz samples: curve (a) is for the natural sample (N) (without additional laboratory irradiation), curve (b) is for the sample that was additionally irradiated 19.2 Gy, curve (c) is the sample was heated to 500 °C.

The absorption spectra of quartz samples are presented in Fig. 10: curve (a) is the natural sample (N) (without additional laboratory irradiation). Two absorption bands are observed in the UV region, a narrow band with pretty strong intensity at 226 nm, and a broad band with weak intensity at 280–400 nm (center at 345 nm). For the irradiated sample (19.2 Gy), besides the band at 226 nm, the absorption spectrum also includes broadband with pretty strong intensity in the range from 280 to 700 nm (curve b). By deconvoluting the absorption spectrum with the energy scale, we observed four separate bands at 5.1 eV (226 nm), 3.6 eV (345 nm), 2.7 eV (450 nm), and 2.1 eV (585 nm). In case the measurement was performed after the sample was heated to 500 °C (before heating, the sample was irradiated by the γ-ray with 5 kGy), the bands in the visible region almost disappear, and the absorption spectrum only consists of two bands with very small intensity at 226 and 345 nm (curve c). Thus, the absorption bands at 345, 450 and 585 nm are related to the irradiation process. In addition, for all samples, the absorption spectra also include a strong absorption band below 250 nm which is the absorption of the SiO_2_ network.

**Fig. 11:**
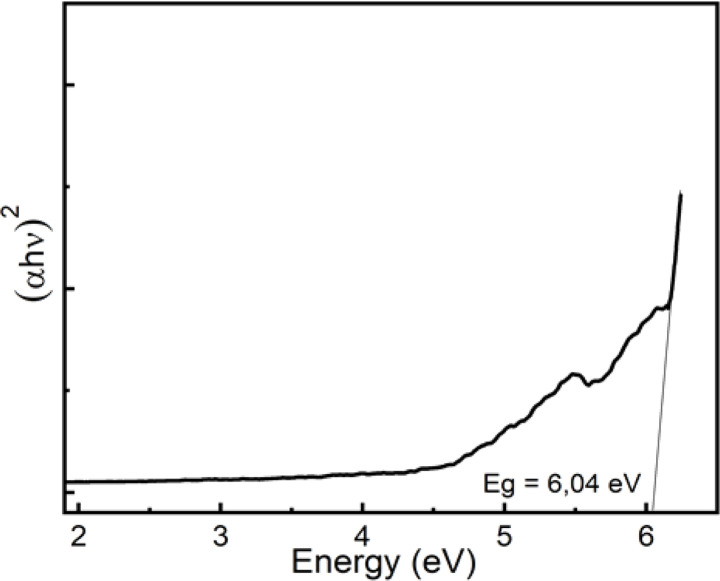
Tauc plot to determine the optical band gap *E*_g_ for the direct allowable transition in natural quartz.

The optical band gap *E*_g_ in crystalline and amorphous materials can be determined from the optical absorption spectrum. Accordingly, near the edge of an absorption curve, the absorption coefficient (*α*) is related to phonon energy (*hν*) by the relationship:

1 *αhν* = *B*(*hν* − *E*_*g*_)^*n*^

where *B* is a constant; *E*_g_ is the optical band gap; *n* is the index that can take on the values of 1/2 or 2 corresponding to direct and indirect transitions, respectively; *α* is the absorption coefficient which relates to absorbance and thickness by the following expression:2
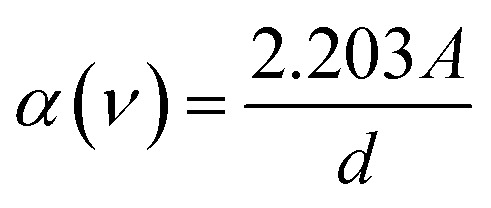
By plotting (*αhν*)^1/*n*^ as a function of *hν*, the band gap energy can be obtained by extrapolating the linear region of the curve to (*αhν*)^1/*n*^ = 0. Using the value *n* of 1/2 is the most appropriate value for the crystalline materials, the dependence of (*αhν*)^2^ on *hν* for natural quartz is indicated in Fig. 11. By extrapolating the linear region of this curve, the value of *E*_g_ was found to be 6.04 eV for the natural sample. The band gap in natural quartz is smaller than that in the pure SiO_2_ crystal sample. This result can be explained by the presence of defects that give rise to energy-localized states. These energy levels are distributed into the bands in the forbidden region of the material. This leads to a reduction in the optical band gap *E*_g_ of direct allowable transition in natural quartz samples.^5^

#### 3.3.2. Properties of the absorption bands

**Fig. 12:**
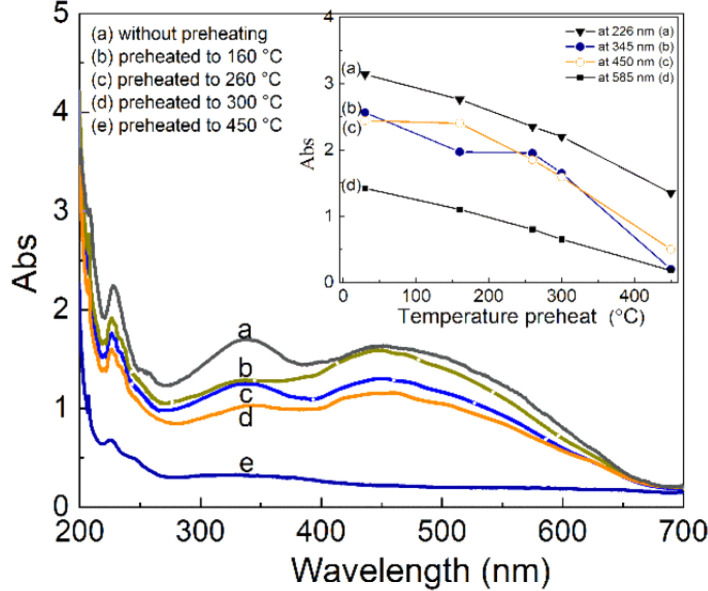
Absorption spectra of natural quartz irradiated with a gamma dose of 19.2 Gy, measured under different preheated conditions (a) no preheated, (b) preheated to 160 °C, (c) preheated to 260 °C, (d) preheated to 300 °C, (e) preheated to 450 °C.

To study the effect of irradiation and heating on the absorption properties, the absorption spectra of the samples were measured under preheated different conditions. In our laboratory, the gamma radiation used for irradiation is generated by the Cobalt PICKER/C9 source with a dose rate of 32 cGy minute^−1^. To quantify the radiation dose, we chose radiation dose rates that are multiples of 0.32 Gy (specifically 6.4 Gy and 19.2 Gy). In addition, because the sensitivity of the TL signal is often much greater than the absorption signal, in the absorption measurements we had to increase the exposure dose 3 times compared to the TL measurements (from 6.4 Gy to 19.2 Gy). For a high dose of gamma radiation, the changes in absorption bands of the samples would be observed clearly. Fig. 12 presents the absorption spectra of quartz samples supplemented irradiation with (19.2 Gy) laboratory dose and preheated at different temperatures. Curve (a) is of the sample preheated at 30 °C, curve (b) is of the sample preheated to 160 °C, curve (c) is of the sample preheated to 260 °C, curve (d) is of the sample preheated to 310 °C and curve (e) is of the sample preheated to 450 °C.

The change in intensity of the absorption bands according to the sample treatment temperature before taking the measurement is described in the inset in Fig. 12. It can be observed that: the absorption band at 262 nm (curve (a)) has the intensity decreased steadily when the sample was preheated at 160 °C, 260 °C, 310 °C and 450 °C, show that this band is related to all electron traps below 450 °C. For the absorption band at 345 nm (curve (b)), the intensity decreases rapidly when the sample was preheated to 160 °C, then the intensity did not change when the sample was preheated to 260 °C. The absorption intensity further decreased when the sample was preheated at 310 °C and 450 °C. This shows that the absorption centers of light at 345 nm is associated with electron traps of less than 160 °C and greater than 300 °C, respectively, with little to do with electron trap at 238 °C. For the absorption band at 450 nm (curve (c)), the absorption intensity changed very slightly when the sample was preheated to 160 °C but decreased rapidly when the sample was preheated at 260 °C, 300 °C and 450 °C. It demonstrates that the absorption band at 450 nm is directly related to the electron traps at 325 °C, and 375 °C.

For the absorption bands at 585 nm (curve (d)), the intensity of the band decreased steadily when the sample was preheated at 160 °C, 260 °C, 310 °C and 450 °C, It demonstrates that the absorption bands at 585 nm related to all electron traps below 450 °C that are produced by irradiation. That can be explained as follows: when the sample is preheated to a certain temperature, the electron traps corresponding to a temperature lower than this temperature will be emptied, which is possible to reduce the concentration of the absorption center at the wavelength that is associated with these electron traps, and so the intensity of the band reduced. The origin and properties of absorption centers in the UV-vis region are discussed as follows: the pure quartz crystal has a rather large optical band gap, so it cannot absorb photons with wavelengths greater than 200 nm.^4,12^ However, real crystals have imperfections in the crystal lattice, stemming either from the intrusion of impurity atoms (see Table 1) or from structural defects. Thus, the absorption of photons with wavelengths larger than 200 nm of this crystal arises from only two sources: the specific defects of the SiO_2_ lattice and impurity atoms present during the formation and growth of quartz crystals.^27^ According to Nunes *et al.*, the absorption band at 226 nm is related to the [AlO_4_]^−^ center.^6^ However, according to some other authors in ref. 5 and 27, this band is related to the presence of 
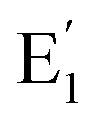
 centers ([SiO_3_]^−^ – oxygen vacancy).

Our experimental results show that the irradiated and heat-treated processes did not change significantly the intensity and position of the absorption band at 226 nm, so it can be inferred that this center is quite thermally stable. In our view, such a thermally stable center can only be related to defects in the SiO_2_ lattice structure. The defects are in the short-range order of the silicate structure and are governed by silicon–silicon and silicon–oxygen interactions. In other words, we also assigned a 226 nm band to the 
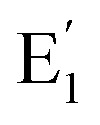
 centers. The absorption bands between 280 nm and 700 nm only occur when the sample is irradiated with γ-rays. Therefore, it is derived from impurity atoms that act as optical centers present during quartz crystal formation. By combining EPR measurements and UV-vis absorption spectroscopy, Nunes *et al.*^6^ and Itoh *et al.*^23^ showed that the absorption band at 345 nm is related to [AlO_4_]^−^ absorption centers produced by irradiation. However, according to Monarumit *et al.*,^5^ this band is likely that be related to the Fe^3+^ ions ([FeO_4_]^−^). Our experimental results show that the absorption band at 345 nm is associated with electron traps at 110 °C and 325 °C (these electron traps have the characteristic of being susceptible to optical bleaching). Therefore, this absorption band is likely related to the [AlO_4_]^−^ center and less to the [FeO_4_]^−^ center.

Studies on the optical absorption properties of natural quartz have shown that the absorption bands in the visible region (at 470 nm and 580 nm) are all related to the [AlO_4_/h^+^]^0^ centers.^1,10,13,23,26^ The formation of [AlO_4_/h^+^]^0^ centers during irradiation is as follows: in the quartz crystal, the Al^3+^ ions that displace Si^4+^ ions are accompanied by interstitial protons (H^+^) or alkali ions (M^+^) to ensure local charge balance. The Irradiation will produce electron/hole pairs, and at the same time release, interstitial ions into the quartz c-channel may also lead to the creation of a hole (h^+^) in a non-bonding p orbital of an oxygen atom adjacent to substitutional aluminum.^1,10,13,23,26^ As a result, [AlO_4_/h^+^]^0^ absorption centers are generated and form absorption bands in the visible light region.^6,32,33^

**Fig. 13:**
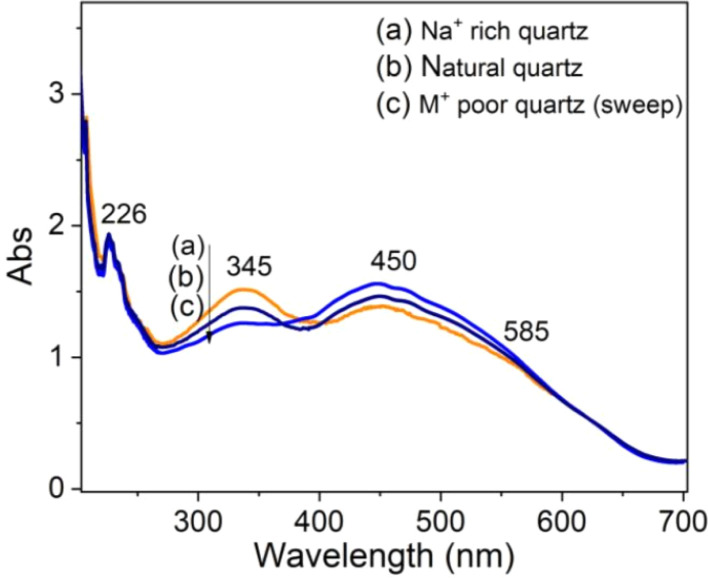
Absorption spectra of natural quartz irradiated with gamma dose of 19.2 Gy (a) Na^+^ rich quartz sample, (b) natural quartz sample and M^+^ poor quartz sample.

To clarify the role of alkali metal ions (M^+^) and hydrogen ions (H^+^) ions in the absorption properties of quartz, we measured the absorption spectra on three types of samples: natural quartz sample, Na^+^ rich sample, and M^+^ poor samples.^9^ The results presented in Fig. 13 show that: (i) absorption bands at wavelengths of 226 nm and 585 nm are unchanged; (ii) the band at 345 nm increases strongly for Na^+^ rich samples and decreases for M^+^ poor samples (H^+^ rich); (iii) the 450 nm band change slightly in intensity. It should also be noted that removing M^+^ ions using electric field and temperature also enriches the sample with H^+^ ions.^9^ These results suggest that the absorption centers at 345 nm and 450 nm are mainly related to charge compensation by M^+^ and H^+^ ions, respectively.

The formation and destruction of absorption centers in colorless quartz mainly involves Al^3+^ ions replacing Si^4+^ ions accompanied by alternating protons (H^+^) or alkaline ions (M^+^) to ensure local charge balance to form [AlO_4_/M^+^(or H^+^)]^0^ centers. Irradiation can create a hole (h^+^) in a non-bonding p orbital of an oxygen atom adjacent to substitutional aluminum to form [AlO_4_/h^+^]^0^ absorption centers. Also, the [AlO_4_/M^+^]^0^ centers can be destroyed to form [AlO_4_]^−^ centers and release M^+^(or H^+^) ions. Thus, the formation of absorption ([AlO_4_/h^+^]^0^ and [AlO_4_]^−^) centers in the region UV-vis can be described by the following diagrams:
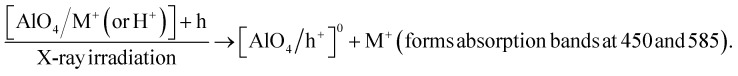

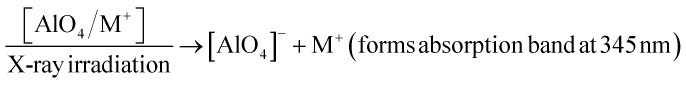


The correct identification of these centers needs a more detailed investigation, which should include ESR spectroscopy studies. Unfortunately, we could not observe these transitions due to experimental limitations. The absorbed dose saturation limit is a concern when calculating age using the quartz technique because the samples used in age calculation must not have reached the saturation state of the environmental irradiation dose.

**Fig. 14:**
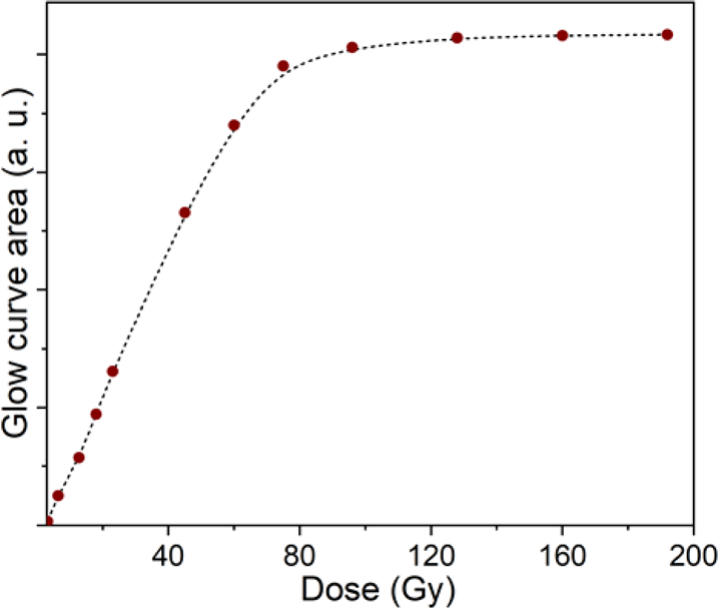
The response of absorption *versus* irradiation dose (area under the curve).

Fig. 14 presents the response of absorption *versus* irradiation dose (the area under the absorbed curve). The response curve was linear over the dose range from 3.2 Gy to 80 Gy. The absorbed dose saturation begins when the irradiation dose is above 80 Gy. This result shows that the natural quartz samples used for our study have not reached a state of irradiation saturation.

#### 3.3.3. The correlation between absorption and thermoluminescence c

In TL materials, energy states localized in the bandgap (near the bottom of the conduction band and the top of the valence band) act as charge traps, they absorb ionizing radiation and participate in the TL process. The change of carrier concentration in the charge traps depends on the nature of the traps as they interact with the radiation or/and the temperature. For colorless natural quartz, if the centers that absorb light in the UV-vis region are formed under the effect of ionizing radiation and participate in the TL process, they will be reduced during heating.

**Fig. 15:**
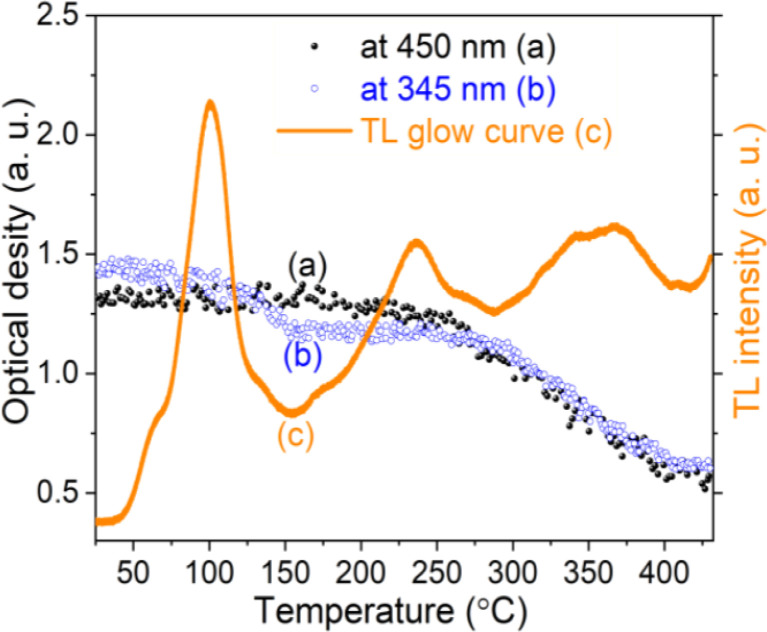
The change in absorption intensity of the 450 nm (a), and the 345 nm (b) bands of the natural quartz sample when increasing the temperature of the sample from RT to 450 °C.

Experimentally, we can use absorption measurements concurrently with TL measurements to test that hypothesis. Instead of having to perform a large set of absorption measurements at different temperatures, we measure the absorption spectrum using monochromatic light (at maximum wavelength in the absorption spectrum), at the same time, the sample is heated.^24,32,34^Fig. 15 (curves a and b) present the change in absorption intensity of the 450 and 345 nm bands, respectively. In these measurements, the temperature of the sample was gradually raised from RT to 430 °C and the sample was previously additionally irradiated with 19.2 Gy. It can be seen that the absorption intensity of the 345 nm band decreases in the temperature ranges of 80–130 °C and 280–410 °C, completely equivalent to the glow peaks at 110 °C, 325 °C and 375 °C on the TL curve (curve c). For the absorption band at 450 nm, the absorption intensity starts to decline when the temperature reaches 170 °C. Compare with the TL glow curve (c) (measurement with the same heating rate), then they are completely equivalent to the glow peaks greater than 200 °C. The above-obtained results can be explained as follows: the theory for the TL process has shown that the TL intensity is determined by the rate of electron depletion captured on the trap or hole in the recombination center:3
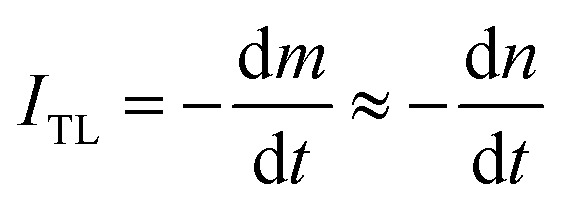
where *m* is the concentration of the hole in the recombination center and *n* is the concentration of the electron in the trap. On the other hand, absorbance is described by the expression *A* = *εdm*; where *ε* is the absorption coefficient, *d* is the thickness of the absorbing medium, and *m* is the absorption center concentration, both *ε* and *d* are constant for a sample. The change in concentration of the absorbed center over time will be:4
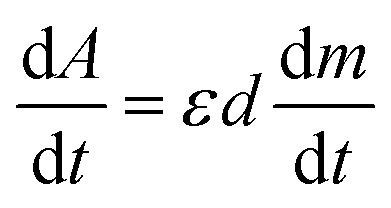


Note that, for monochromatic light, absorption occurs only at centers with suitable physical properties (location and nature of the center), then can combine (3) and (4) we have:5
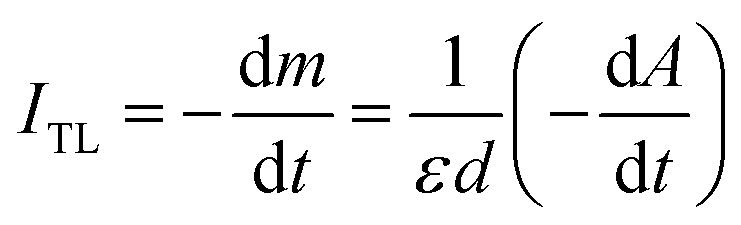


If in both measurements we heat the sample at the same constant heating rate, then eqn (5) becomes:6
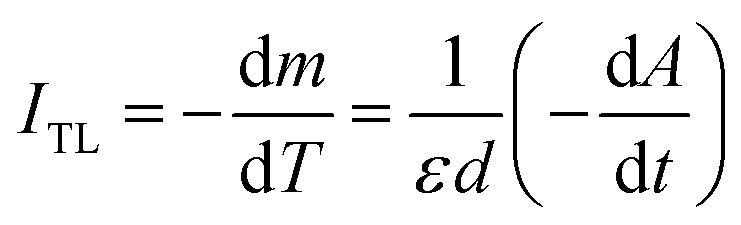


Eqn (6) shows that the TL signal intensity is proportional to the rate of decline in the concentration of the absorption centers for a monochromatic light during sample heating.^35–39^ In this experiment, at the initial time of heating, the carrier concentration in the absorption centers is greatest, so the absorption intensity reaches the maximum value for a monochrome light. As the temperature increases, the charges on the traps are released resulting in a decrease in the absorbing center concentration, and a decrease in absorption band strength is observed. These experimental results show that the TL process occurs concurrently with the reduction of the absorbent center produced in the irradiation process.

#### 3.3.4. The correlation between absorption and optical bleaching

**Fig. 16:**
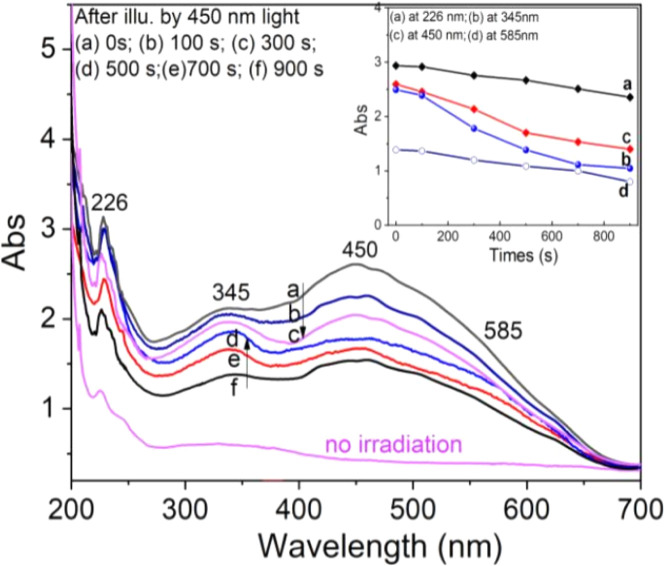
The intensity-response of absorption bands at 226 nm, 345 nm, 450 nm and 585 nm over time of illumination with blue light (450 nm) for different periods. The inset is the decrease in intensity of the absorption bands with a time of illumination by 450 nm light.

The effect of optical bleaching (OB) on the intensity of absorption bands is shown in Fig. 16. After irradiation by γ-rays at room temperature, the samples were illuminated with blue light (450 nm) for periods of 100 s, 300 s, 500 s, 700 s, and 900 s. Then, absorption spectra were recorded in the wavelength range from 200 nm to 700 nm. The results show that there was a change in absorption intensity but the position of peaks is not affected. The intensity of the absorption band at 226 nm was slightly reduced with the illumination time (curve a). For the absorption band at 345 nm, its intensity decreases slowly in the first 100 s (curve b) and then decreases rapidly with further light exposure (200 to 900 s). For the absorption bands at 450 nm and 585 nm, its intensity decreases rapidly in the first 100 s (curve c, d) and then decreases slowly with further light exposure (200 to 900 s). These results show that the absorption band at 226 nm is less influenced by the bleaching due to the blue light in comparison with other bands.

Since the absorption bands at 345 nm and 450 nm are both involved in electron traps at 110 °C, 238 °C, 325 °C and 375 °C, the attenuation of the absorption intensities of these bands may be related to the photosensitive traps at 110 °C and 325 °C.^17,19,20,39^ These experimental results can be explained as follows: when the sample is exposed to blue light, electrons in photosensitive traps (at 110 °C and 325 °C) can be released, and then recombine with the hole trapped at the [AlO_4_/h^+^]^0^ centers, this reduces the absorption centers [AlO_4_/h^+^]^0^ generated by previous irradiation, *i.e.* the absorption intensity of the bands decreased. On the other hand, since during the first 100 s of illumination, electrons released from deep traps (*e.g.* traps at 325 °C) can be retrapped at 110 °C traps, this decelerated the degradation [AlO_4_]^−^ absorption centers, so band intensity at 345 nm and 450 nm decreases slowly.^33,35,39–43^ In summary, the process of reduction of absorption centers in the UV-vis ([AlO_4_/h^+^]^0^ and [AlO_4_]^−^) region can be described as follows:
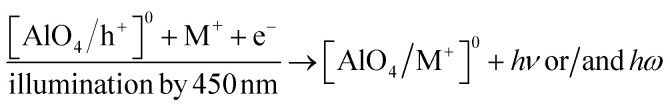

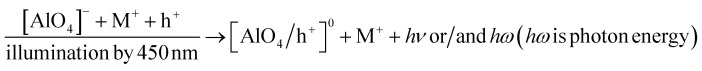


## 4. Conclusion

The TL properties of colorless natural quartz were studied in detail through measurements of TL, TSC and OA. The properties of the TL peaks and the emission bands in TL spectrum as well as the relationship between them were determined. Using the initial rise and isothermal decay method, the dynamics parameters including the activation energy, lifetime, and frequency factor were calculated for all glow peaks. The experimental results on TSC are proofs of the recombination mechanism with the participation of the energy bands in the TL process. The role of irradiation and heat treatment in the formation of absorption centers and their relationship to different optically sensitive and thermostable charge traps were studied. The role of alkali metal ions (M^+^) and hydrogen ions (H^+^) ions in the absorption properties of quartz were discussed. The absorption center at wavelength 345 nm is mainly related to charge compensation by M^+^ ions (less related to H^+^ ions), while the absorption centers at wavelength the 450 nm and 585 nm waves are related to both M^+^ and H^+^ ions. The correlation between the OA process along with TL and bleaching time by blue light was also studied in detail through the intensity attenuation of absorption bands in the visible region and TL peaks. The observed results on the correlation between OA, TL, and OB all show: that the TL process occurs concurrently with the reduction (bleach) of the absorbent centers produced in the irradiation process. The TL signal saturation are observed for at 110 °C peak when the irradiation dose is above 22 Gy. For the at 238 °C peak, saturation appear when the irradiation dose is above 45 Gy and above 80 Gy for the two peaks at 325 °C and 375 °C. In the dose range from 0 kGy to 80 Gy, the absorbance (the area under the absorbed curve) responded almost linearly to the irradiation dose. The absorbed dose saturation begins when the irradiation dose is above 80 Gy.

## Data availability

The data supporting this study's findings are available on request from the corresponding author [Phan Van Do, email: phanvando@tlu.edu.vn]. The data are not publicly available due to [reason, privacy].

## Conflicts of interest

There are no conflicts to declare.
